# Single-arm, open-label pilot intervention study to investigate an effect of oral 5-aminolevulinic acid plus sodium ferrous citrate on glucocorticoid reduction in patients with adult-onset Still disease: Study protocol for clinical trial (SPIRIT compliant)

**DOI:** 10.1097/MD.0000000000022708

**Published:** 2020-12-11

**Authors:** Remi Sumiyoshi, Tomohiro Koga, Toshimasa Shimizu, Shuntaro Sato, Shigeki Tashiro, Naoki Hosogaya, Hiroshi Yamamoto, Atsushi Kawakami

**Affiliations:** a Department of Immunology and Rheumatology, Division of Advanced Preventive Medical Sciences, Nagasaki University Graduate School of Biomedical Sciences; b Nagasaki University Hospital, Clinical Research Center; c Center for Bioinformatics and Molecular Medicine, Nagasaki University Graduate School of Biomedical Sciences, Nagasaki, Japan.

**Keywords:** 5-aminolevulinic acid/sodium ferrous citrate, adult-onset Still disease, glucocorticoid, heme oxygenase-1, open-label

## Abstract

**Background:**

Glucocorticoids are an important class of medication for patients with adult-onset Still disease (AOSD), however, relapse following glucocorticoid reduction and adverse events due to long-term effects of glucocorticoid are still problematic. It is of course essential to minimize the risk of treatment. Immunosuppressive therapies such as methotrexate and biologics including tocilizumab are used in glucocorticoid-dependent patients with AOSD, but no second-line treatments for patients with glucocorticoid dependence have been established yet. Given that these drugs also have the potential to cause adverse events, alternative treatments are sought. Recently, elevated heme oxygenase-1 (HO-1) has been reported in the serum of patients with AOSD, suggesting that HO-1 activity contributes to AOSD pathogenesis and may represent a new therapeutic target for the treatment of AOSD. The amino acid 5-aminolevulinic acid (5-ALA) is a non-proteinogenic δ amino acid in human body. An addition of ferrous iron to 5-ALA enhances heme biosynthesis. The increase in heme in vivo induces HO-1 production, a heme-degrading enzyme. Elevated HO-1 has been suggested to contribute to the pathogenesis of AOSD, and administration of 5-ALA and ferrous iron may be a potential treatment for AOSD.

**Methods/design:**

This study is a single-arm, open-label pilot intervention study using clinical endpoints to investigate the effects of oral 5-ALA with sodium ferrous citrate on glucocorticoid reduction in patients with AOSD receiving glucocorticoid therapy.

**Discussion:**

This pilot intervention study will provide evidence regarding the effectiveness and safety of 5-ALA/sodium ferrous citrate as a potential new therapeutic agent for glucocorticoid-dependent patients with AOSD.

**Trial registration:**

This study was registered in the Japan Registry of Clinical Trials (https://jrct.niph.go.jp) on January 14, 2020 as jRCTs071190042.

## 1 Introduction

Adult-onset Still disease (AOSD) is a systemic inflammatory disorder of unknown etiology, characterized by fever, joint symptoms, and skin rash.^[1]^ Initial treatment for patients with moderate to severe disease activity is systemic administration of moderate to high doses of glucocorticoids, however, response varies among patients. Furthermore, cases exist of patients becoming resistant to glucocorticoids and who relapse during tapering of doses. Adverse events such as organ damage and infection associated with long-term administration may be a problem for patients treated with glucocorticoid alone. In such cases, combination therapy with tocilizumab, methotrexate, and/or cyclosporine is an option. Clinical practice guidelines for adult Still disease in Japan^[2]^ include recommendations for these treatments, but evidence regarding safety and efficacy is scarce, and treatments are still under development.

Although AOSD pathogenesis remains unclear, elevated heme oxygenase-1 (HO-1) has recently been reported in the serum of patients with AOSD. Moreover, its expression decreases following treatment in parallel with serum ferritin levels, which are conventionally used as a marker of disease activity in AOSD.^[3]^ This finding suggests that HO-1 activity contributes to AOSD pathogenesis.

The amino acid 5-aminolevulinic acid (5-ALA), a non-proteinogenic δ amino acid found in the human body, promotes intracellular energy metabolism, and addition of ferrous iron to 5-ALA enhances heme biosynthesis. The increase in heme in vivo induces HO-1 production, a heme-degrading enzyme. The increase in heme also induces NF-E2 related factor (Nrf-2) expression, upstream of HO-1, and its downstream anti-inflammatory cytokines, which exert immune tolerance and anti-inflammatory effects.^[4]^ It has been suggested that elevated HO-1 in patients with AOSD results from an excessive immune response. Therefore, oral 5-ALA with sodium ferrous citrate (SFC) may be effective in AOSD treatment, exerting immune tolerance, and anti-inflammatory effects via induction of HO-1 production.

We hypothesized that additional 5-ALA/SFC administration in patients with AOSD may improve disease activity and allow for glucocorticoid reduction. We; therefore, designed this study as the first step in validating the effectiveness of 5-ALA/SFC in the treatment of AOSD. Herein, we describe the final study protocol (version 1.4; May 19, 2020). The results of this study are expected to provide evidence on the usefulness of 5-ALA/SFC in the treatment of glucocorticoid-dependent patients with AOSD.

## 2 Methods/design

### 2.1 Study design

The present study design is in accordance with Standard Protocol Items: Recommendations for Interventional Trials and Consolidated Standards of Reporting Trials 2010 guidelines.^[5,6]^ This is a single-arm, open-label, pilot intervention study to investigate the effects of 5-ALA/SFC on glucocorticoid reduction in patients with AOSD.

The study will be conducted at 2 centers in Japan. In total, 7 patients with AOSD will be recruited to the study, with a 16 week duration of intervention. The study has been approved by the certified review board (CRB) of Nagasaki University (CRB approval no.: CRB19-017) and is registered in the Japan Registry of Clinical Trials (https://jrct.niph.go.jp) as jRCTs071190042. The study will be conducted in accordance with the principles of the Declaration of Helsinki,^[7]^ the Clinical Trials Act (Act No. 16 of April 14, 2017), the Act on the Protection of Personal Information and related regulatory notifications, and the present study protocol.

### 2.2 Participant recruitment

Participants will be recruited at the Nagasaki University Hospital and Nagasaki Genbaku Hospital. The study will be explained to participants by their attending rheumatologist; participants will then be asked to voluntarily sign an informed consent form before their participation.

### 2.3 Inclusion criteria

Inclusion criteria are as follows:

(1) Patients aged 20 years or older at the time of consent.
(2) Patients undergoing treatment in an outpatient setting.
(3) Patients diagnosed with AOSD according to Yamaguchi criteria.^[8]^
(4) Patients treated with oral prednisolone (PSL) at a dose of between 5 mg/d and 30 mg/d.
(5) Patients who continued the same dose of oral PSL for at least 8 weeks before 5-ALA/SFC administration.
(6) Patients who have not received a change in dose or any new treatment for AOSD for at least 8 weeks before 5-ALA/SFC administration.
(7) Patients who are willing and able to give written informed consent and comply with requirements of the study protocol.

### 2.4 Exclusion criteria

Exclusion criteria are as follows:

(1) Patients classified as severe according to the AOSD severity classification of the Research Team for Autoimmune Diseases, the Research Program for Intractable Disease of the Ministry of Health, Labor and Welfare, Japan.^[9]^
(2) Patients who have previously taken 5-ALA/SFC.
(3) Patients who cannot use appropriate contraception during the drug administration period of the study.
(4) Female patients during pregnancy or lactation.
(5) Patients judged inappropriate for any other reason by the clinical investigator or clinical trial physician.

### 2.5 Study protocol

**Figure 1:**
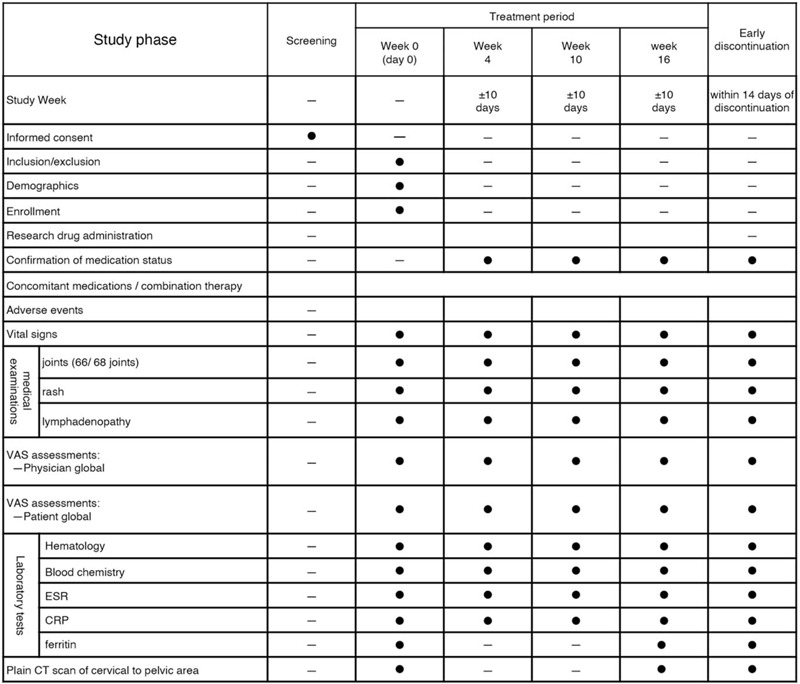
Treatment schedule and outcome measures. CRP = C reactive protein, ESR = erythrocyte sedimentation rate, VAS = visual analog scale.

AOSD patients who have received a consistent dose of PSL for at least 8 weeks will be administered an additional daily 200 mg of 5-ALA/SFC for 16 weeks. At weeks 4 and 10 after the start of study, the oral PSL dose will be reduced according to the prescribed protocol to determine whether relapse or exacerbation occur. A physician will explain the study protocol to each patient and, if consent is obtained, the physician will perform the observation/examination at the time of registration in keeping with the description in Figure 1. According to the inclusion and exclusion criteria, the physician will fax the participant registration form to the registration center.

After the visit date, the physician will continue to administer 5-ALA/SFC and conduct necessary examinations and surveys until week 16 in accordance with the schedule in Figure 1. The protocol for PSL reduction is as follows, depending on PSL dose at the time of visit.

1. For 15 mg < PSL ≤ 30 mg/day: 8% to 12% dose reduction.
2. 7.5 < PSL ≤ 15 mg/d: 1 mg/d dose reduction.
3. For PSL ≤ 7.5 mg/d: 0.5 mg/d dose reduction.

For example, a patient taking PSL 8 mg/d at the start of the study should be reduced to 7 mg/d at week 4 and 6.5 mg/d at week 10.

During the study period, new initiation of the following treatments is prohibited: the administration of immunosuppressants, biologics or Janus kinase inhibitors, intra-articular corticosteroid injections at joints, and oral nonsteroidal anti-inflammatory drug and suppositories.

A patient may be discontinued prematurely for the following reasons (patients who discontinue before completing the trial will not be replaced):

- The patient asks to leave the trial.
- Continuing participation is inadvisable due to adverse event(s).
- 5-ALA/SFC must be withdrawn more than 4 weeks for some reason(s).
- 5-ALA/SFC must be reduced more than half the daily dose for some reason(s).
- In the physician's opinion, continuation in the trial would be detrimental to the patient's well-being.

### 2.6 Adverse events

A serious adverse event is defined as any untoward medical event that occurs at any dose, results in death, is life-threatening, requires inpatient hospitalization or prolongation of existing hospitalization, results in persistent or significant disability or incapacity, or causes a congenital anomaly or birth defect.

All serious adverse events occurring between the signing of informed consent and the end of the trial will be documented in the medical records and reported to the CRB by the responsible investigator in accordance with Japanese regulations.

### 2.7 Study endpoints

#### 2.7.1 Primary endpoint

The primary endpoint is the proportion of patients who were able to maintain PSL reduction without relapse or exacerbation after study initiation.

#### 2.7.2 Secondary endpoints

The secondary endpoints are as follows:

(1) Clinical signs (fever, joint symptoms, rash, and lymph node enlargement): change from baseline at 4, 10, and 16 weeks, and at the time of drug discontinuation.
(2) Abnormalities in laboratory tests based on the systemic feature score^[10,11]^ (white blood cells [/μL], hemoglobin [g/dL], platelet [/μL], C reactive protein [mg/dL], and erythrocyte sedimentation rate [mm/h]): change from baseline at 4, 10, and 16 weeks, and at the time of drug discontinuation.
(3) Changes from baseline systemic feature score at 4, 10, and 16 weeks, and at the time of drug discontinuation.
(4) Physician global assessment (disease activity assessment, 100 mm visual analog scale): changes from baseline at 4, 10, and 16 weeks, and at the time of drug discontinuation.
(5) Patient global assessment (disease activity assessment, 100 mm visual analog scale): changes from baseline at 4, 10, and 16 weeks, and at the time of drug discontinuation.
(6) Serum ferritin levels: changes from baseline at 4, 10, and 16 weeks, and at the time of drug discontinuation.

### 2.8 Data collection and management

Appropriate and authorized persons (investigators, physicians, and collaborators) will prepare a case report form (CRF). All data recorded in the CRF must be consistent with original material unless data recorded directly in the CRF are used as the source material. The investigator will collect data at each visit during the study, in accordance with the schedule in Figure 1. All study findings and documents will be made confidential and patients will always be identified by their patient number and/or birth date, never by name. Confidentiality patient-identifying documents will be maintained by the investigator to preserve participant anonymity.

During the study, a sponsor-investigator will undertake regular site visits to review protocol compliance, conduct source data verification, assess drug accountability and management and assess laboratory procedures to ensure that the study is being conducted according to all pertinent requirements.

### 2.9 Statistical analysis

The population to be analyzed is defined as all subjects receiving at least 1 dose of 5-ALA/SFC throughout the study. Characteristics of all 5-ALA/SFC-treated populations will be summarized using descriptive statistics. Effectiveness endpoint listed in 2.7 will be estimated point estimates and 95% confidence intervals. Safety and tolerability analyses will be based on this analysis set. Adverse events will be stratified by serious vs. nonserious, causal relationship, severity, outcome, and presence or absence of clinical relapse. Analyses will be categorized and tabulated by name and a list will be formulated, including causality, extent, and outcomes.

Data relevant to this clinical trial will be analyzed using R software version 4.0.0 or higher, or JMP Pro 15 or higher.

## 3 Discussion

AOSD is a systemic inflammatory disorder. Autoantibodies such as antinuclear antibodies are generally not detected, however, it is thought that hypercytokinemia involving interleukin-1β (IL-1β), IL-6, IL-18, and tumor necrosis factor-α, due to abnormal activation of innate immune cells such as monocytes and macrophages, may be involved in disease pathogenesis.^[12]^ The clinical course of AOSD is divided into 3 types: monophasic, intermittent, and chronic.^[13–15]^ Long-term treatment is particularly essential in intermittent and chronic types in which systemic symptoms relapse repeatedly. As mentioned previously, there is still no established, well-evidenced treatment for AOSD. Treatment options consist of glucocorticoids, biologics, and immunosuppressive drugs, but adverse events and concomitant infections associated with long-term medication can be problematic. Treatment options with different mechanisms of action from those above are therefore sought to solve these problems.

HO-1, also known as heat shock protein-32, is a 32 kD heme-degrading enzyme^[16]^ induced by various stresses which is reportedly increased in the serum of patients with adult-onset Still disease. Heterodimers of Nrf-2 and small Maf, transferred from the cytoplasm to the nucleus by stress, bind to antioxidant responsive elements and induce the transcription of antioxidants including HO-1 and ferritin.^[17]^ Induction of HO-1 production is thought to elevate expression of regulatory dendritic cells, induce regulatory T-cell differentiation and immune tolerance, promote M2 macrophage differentiation and exert anti-inflammatory effects.^[18]^

The addition of divalent iron to 5-ALA enhances heme biosynthesis.^[18]^ In vivo increases in heme induce HO-1 production, which is a heme-degrading enzyme. These increases also induce Nrf-2, upstream of HO-1, and its downstream anti-inflammatory cytokines, which exert immune tolerance and anti-inflammatory effects. Oral administration of 5-ALA/SFC may be an effective treatment for AOSD via the immune tolerizing and anti-inflammatory effects of HO-1.

In the present study, we will administer 5-ALA/SFC to glucocorticoid-dependent patients with AOSD, and determine whether PSL reduction can be maintained without relapse or exacerbation after study initiation. There are no restrictions on concomitant use of immunosuppressive drugs or biologics, and this is a pilot study examining the steroid reduction effect of addition of 5-ALA/SFC to standard treatment.

In our study, we will use the systemic feature score as a secondary endpoint to determine clinical effectiveness of 5-ALA/SFC. This scoring system, consisting of 5 clinical and 5 laboratory assessments, was designed to evaluate systemic disease features.^[10]^ It was also previously used as a secondary endpoint in a phase III trial on the efficacy of tocilizumab in Japanese patients with AOSD.^[11]^ Because the present study excludes patients receiving high dose of glucocorticoids and patients with severe AOSD, the systemic feature score at baseline is expected to be low. As this score is likely to be elevated at the time of relapse, we considered this score be a reasonable endpoint for determining relapse.

The dosage of the investigational drug will be set at 100 mg orally twice daily (200 mg/d) in accordance with a previous study conducted in patients with type 2 diabetes (ClinicalTrials.gov NCT02481141),^[19]^ which demonstrated the safety and tolerability of such a dose.^[19]^ We consider that this dose is acceptable and effective for patients with AOSD.

This trial will evaluate the effectiveness and safety of 5-ALA/SFC in glucocorticoid-dependent patients with AOSD. It will support the potential effectiveness of 5-ALA/SFC in preventing relapse or exacerbation after the reduction of glucocorticoid and in improving patient quality of life and vital prognosis. The findings of this study are expected to provide new treatment options for AOSD.

## Acknowledgments

The authors would like to thank our colleagues and staff at the Rheumatology Department of Nagasaki University Hospital for their support.

## Author contributions

**Conceptualization:** Remi Sumiyoshi, Tomohiro Koga, Toshimasa Shimizu, Naoki Hosogaya, Hiroshi Yamamoto.

**Methodology:** Shuntaro Sato, Shigeki Tashiro.

**Writing – original draft:** Tomohiro Koga, Shuntaro Sato.

**Writing – review & editing:** Remi Sumiyoshi, Toshimasa Shimizu, Shigeki Tashiro, Naoki Hosogaya, Hiroshi Yamamoto, Atsushi Kawakami.
